# Incomplete Restoration of Angiotensin II - Induced Renal Extracellular Matrix Deposition and Inflammation Despite Complete Functional Recovery in Rats

**DOI:** 10.1371/journal.pone.0129732

**Published:** 2015-06-10

**Authors:** Anne-Roos S. Frenay, Saleh Yazdani, Miriam Boersema, Anne Marijn van der Graaf, Femke Waanders, Jacob van den Born, Gerjan J. Navis, Harry van Goor

**Affiliations:** 1 Department of Pathology and Medical Biology, University Medical Center Groningen and University of Groningen, Groningen, the Netherlands; 2 Department of Nephrology, University Medical Center Groningen and University of Groningen, Groningen, the Netherlands; 3 Department of Obstetrics and Gynecology, University Medical Center Groningen and University of Groningen, Groningen, the Netherlands; University of Louisville, UNITED STATES

## Abstract

Some diseases associated with a temporary deterioration in kidney function and/or development of proteinuria show an apparently complete functional remission once the initiating trigger is removed. While it was earlier thought that a transient impairment of kidney function is harmless, accumulating evidence now suggests that these patients are more prone to developing renal failure later in life. We therefore sought to investigate to what extent renal functional changes, inflammation and collagen deposition are reversible after cessation of disease induction, potentially explaining residual sensitivity to damage. Using a rat model of Angiotensin II (Ang II)-induced hypertensive renal disease we show the development of severe hypertension (212 ± 10.43 vs. 146 ± 1.4 mmHg, p<0.001) and proteinuria (51.4 ± 6.3 vs. 14.7 ± 2.0 mg/24h, p<0.01) with declined creatinine clearance (2.0 ± 0.5 vs. 4.9 ± 0.6 mL/min, p<0.001) to occur after 3 weeks of Ang II infusion. At the structural level, Ang II infusion resulted in interstitial inflammation (18.8 ± 4.8 vs. 3.6 ± 0.5 number of macrophages, p<0.001), renal interstitial collagen deposition and lymphangiogenesis (4.1 ± 0.4 vs. 2.2 ± 0.4 number of lymph vessels, p<0.01). Eight weeks after cessation of Ang II, all clinical parameters, pre-fibrotic changes such as myofibroblast transformation and increase in lymph vessel number (lymphangiogenesis) returned to control values. However, glomerular desmin expression, glomerular and periglomerular macrophages and interstitial collagens remained elevated. These dormant abnormalities indicate that after transient renal function decline, inflammation and collagen deposition may persist despite normalization of the initiating pathophysiological stimulus perhaps rendering the kidney more vulnerable to further damage.

## Introduction

Virtually all progressive renal diseases, independent of their primary origin [1], lead to end-stage renal fibrosis. Next to this, aging is also a risk factor in the development of kidney disease [2,3]. End-stage renal disease (ESRD) is a life-threatening condition eventually demanding dialysis or renal transplantation. Renal fibrosis is characterized by excessive accumulation of extracellular matrix components and macrophages in the renal interstitial space [4]. Matrix deposition leads to deterioration of the normal architecture of the kidney, thereby hampering crucial functional aspects [1]. Pharmacological intervention with e.g. inhibitors of the renin-angiotensin-aldosterone system (RAAS) aims at cessation or retardation of the progressive nature of renal fibrosis [5–7]. Under certain conditions during which the kidney suffers, for a limited period of time, from the consequences of hypertension and develops proteinuria there appears to be a remarkable renal capacity for self-repair. After the initial trigger has been removed the kidneys regain their normal functional capacity. For example, in patients with acute kidney injury (AKI), which is characterized by an abrupt decline in renal function, complete renal recovery can be observed. However, recent data indicate that these patients are more vulnerable for the development of renal disease later in life [8–11]. This raises the question whether or not the repair mechanisms after short-term renal disease have been incomplete, thereby resulting in increased vulnerability to subsequent detrimental triggers.

Ang II infusion is a widely employed experimental model to study transient renal disease [12–14]. The model is characterized by severe hypertension, followed by massive proteinuria and with, at the structural level, glomerular and interstitial changes including extracellular matrix deposition and macrophage infiltration. After withdrawal of Ang II infusion, blood pressure returns to control values and proteinuria regresses spontaneously and completely. The model more or less mimics human conditions in which the kidney is suffering from hypertension and/or proteinuria for a limited period of time.

In the present study we aimed at providing better insight into the processes of incomplete recovery after initial damage to the kidney, whilst clinical parameters show complete restoration. For that purpose rats were transiently treated with Ang II via osmotic minipumps.The pumps were removed and the rats were studied for functional and structural parameters for an extended period of time thereafter.

## Materials and Methods

### Animal study and design

Twenty male Wistar rats (240–280 gram, Harlan, Horst, the Netherlands) were housed at the animal research facility under standard conditions with a 12h light-dark cycle and free access to drinking water and standard rat chow. After one week of acclimatization to the local vivarium and two weeks of training for daily blood pressure measurement, osmotic minipumps (model 2004, Alzet, Cupertino, CA, USA) were placed intraperitoneally via an abdominal midline incision under general anesthesia (2% Isoflurane/O_2_) to infuse either Ang II (435 ng/kg/min, n = 10; Sigma-Aldrich Corporation, St. Louis, MO, USA) or vehicle (0.9% NaCl, n = 10). After three weeks of continuous infusion, pumps were removed under general anesthesia. In the same procedure an open kidney biopsy was obtained, to determine the extent of renal structural changes. In this way every rat served as its own control. After surgical removal of a small part of the upper pole from the left kidney, gelfoam was applied to achieve hemostasis. Post-operatively, all rats received one (after pump implantation) or three (after biopsy) subcutaneous injection(s) of 50 μg/kg buprenorphine (Schering-Plough, Houten, the Netherlands) for analgesia and were allowed to recover from surgery at 37°C in a ventilated incubator. Of the former Ang II infused rats, 7 out of 10 rats survived the kidney biopsy procedure and survived till the end of the experiment, whereas this was 10 out of 10 for control rats. The loss of Ang II rats was a consequence of failed hemostatis after taking kidney biopsies under severe hypertensive conditions.

On a weekly basis, body weight was measured and rats were placed in metabolic cages for collection of 24h-urine. Blood samples were collected at baseline, during the kidney biopsy, 4 weeks after biopsy and at termination of the experiment. Urinary concentration of protein was determined using the pyrogallol red molybdate method [15]. Plasma and urine creatinine, urea and sodium levels were determined on the Roche Modular platform (Roche, Diagnostics GmbH, Mannheim, Germany) according to routine procedures in our clinical chemical laboratory (UMCG). During infusion, systolic blood pressure was measured weekly in conscious animals using the tail-cuff method (Apollo 179; IITC Life Science, Woodland Hills, California, USA). The mean of three consecutive measurements was taken as the final value. At termination, eight weeks after cessation of Ang II or 0.9% NaCl infusion, rats were anesthetized (2.0% isoflurane in O_2_). The aorta was cannulated and blood samples were taken. Kidneys were harvested after perfusion with 0.9% NaCl. Coronal kidney slices were fixed in 4% paraformaldehyde and paraffin embedded for immunohistochemical analysis or immediately snap frozen in liquid nitrogen and stored at -80°C for molecular analysis. Experimental procedures were performed in accordance with institutional and national regulations and approved by the Institutional Animal Care and Use Committee of the University of Groningen (IACUC-RuG; DEC number: 6062A).

### Qualitative Real-Time Polymerase Chain Reaction

Quantitative real-time PCR (q-PCR) was performed for renin, OPN, Kim-1, α-SMA, Collagen I, Collagen III, Collagen IV, Collagen V, TGF-beta and Podoplanin. RNA was extracted from snap frozen kidney tissue, containing both cortex and medulla, using the TRIzol method (Invitrogen, Carlsbad, CA, USA). RNA concentrations were measured by a nanodrop UV-spectrometer (Nanodrop Technologies, Wilminton, DE, USA). Complementary DNA (cDNA) was synthesized using Superscript II RT with random hexamer primers (Invitrogen, Carlsbad, CA, USA). Gene expression levels (Applied Biosystems, Foster City, CA, USA) were measured by qualitative realtime-PCR (qRT-PCR) based on the Taqman methodology. HPRT was used as a housekeeping gene with the following primers (Integrated DNA Technologies, Coralville, IA, USA) and probe (Eurogentec, Maastricht, the Netherlands): Forward: 5'-GCC CTT GAC TAT AAT GAG CAC TTC A-3’, Reverse: 5'-TCT TTT AGG CTT TGT ACT TGG CTT TT-3’ and Probe: 6-FAM 5'-ATT TGA ATC ATG TTT GTG TCA TCA GCG AAA GTG-3' TAMRA. The other primers were obtained from Applied Biosystems as Assays-on-Demand (AOD) gene expression products. The AOD ID’s used were: Renin Rn00561847_m1, SPP1 (OPN) Rn00563571_m1, Havcr1 (Kim-1) Rn00597703_m1, Acta2 (α-SMA) Rn01759928_g1, Col1a1 (Collagen I) Rn01463648_m1, Col3a1 (Collagen III) Rn01437683_m1, Col4a1 (Collagen IV) Rn01482927_m1, Col5a1 (Collagen V) Rn00593170_m1, TGF-β1 (TGF-beta) Rn00572010_m1 and PDPN (Podoplanin) Rn00571195_m1. The qPCR reaction mixture contained 20 ng cDNA template and 5 ul mastermix. Nuclease-free water was added to a total volume of 10 μl. All assays were performed in triplicate. The thermal profile was 15 minutes at 95°C, followed by 40 cycles of 15 seconds at 95°C and 1 minute at 60°C. Consequently, the gene expression was normalized by calculating the difference in Ct from the Ct of the reference gene HPRT (∆ CT). The average Ct-values for the target genes were subtracted from the average housekeeping gene Ct-values to yield the delta Ct, and results were expressed as 2-ΔCt.

### Immunohistochemistry (IHC) and immunofluorescence

For immunostaining, sections were stained with periodic acid-schiff (PAS) and evaluated for focal segmental glomerular sclerosis (FSGS). Furthermore, we used primary antibodies for Kidney Injury Molecule 1 (Kim-1) (rabbit anti-Kim-1 peptide 9, 1:400, gift V. Bailly, Biogen Inc, Cambridge, MA, USA), osteopontin (OPN) (mouse anti-OPN, clone MPIIIB10, 1:300, Developmental Hybridoma Studies Institute, Iowa City, IA, USA), macrophages (mouse anti-CD68 ED1, MCA341R AbD, 1:750, Serotec Ltd, Oxford, UK), Desmin (mouse anti-desmin, NCL-DES-DER11, 1:500, Novocastra, Rijswijk, the Netherlands), α-Smooth Muscle Actin (mouse anti-SMA, clone 1A4 A2547, 1:10.000, Sigma, Zwijndrecht, the Netherlands), Collagen I (goat anti-type 1 Collagen, 1310-01, 1:100, Southern Biotech, Birmingham, AL, USA), Collagen III (goat anti-type 3 Collagen, 1330-01, 1:75, Southern Biotech, Birmingham, AL, USA), Podoplanin (rabbit anti-Podoplanin, 11–035, 1:100, Angio Bio, Del Mar, CA, USA) and rat endothelial cell antigen-1 (RECA-1) (mouse anti-RECA-1, HM3012, 1:50, HyCult Biotechnology, the Netherlands) to perform IHC. To ensure specificity of our Podoplanin antibody for lymph vessels (LVs) only, we performed an immunofluorescent (IF) double staining with primary antibodies for Podoplanin (same concentration as IHC), LYVE-1 (rabbit anti-LYVE-1, 1:300, gift Prof. D. Jackson, John Radcliffe University Hospital, Oxford, UK) and VEGFR-3 (goat anti-VEGFR-3, AF743, 1:40, R&D system Inc, Minneapolis, MN, USA). First, sections were deparaffinized and washed with phosphate buffered saline (PBS, pH 7.4). For Kim-1, OPN, ED1, desmin and α-SMA, overnight antigen retrieval was performed in 0.1 M Tris/HCl buffer, pH 9.0, at 80°C. For Collagen I, Collagen III, and Podoplanin antigen retrieval was performed using a citrate, pH 6.0, an EDTA, pH 8.0, and an EDTA, pH 9.0 buffer, respectively. The solution was heated for 15 minutes in a microwave oven at maximum power. Sections were again washed with PBS and endogenous peroxidase was blocked by incubation with 0.3% H_2_O_2_ in PBS for 30 minutes. Then, sections were incubated with primary antibodies for 60 minutes at room temperature. Binding was detected using sequential incubation with primary antibodies associated peroxidase-labeled secondary and tertiary antibodies (Dakopatts, Glostrup, Denmark) for 30 minutes. All antibodies were diluted with PBS containing 1% Bovine Serum Albumin (BSA) and 1% normal rat serum was added to the secondary and tertiary antibody dilutions. Peroxidase activity was developed using 3,3’-diaminobenzidine tetrachloride (DAB) for 10 minutes containing 0.03% H_2_O_2_. Counterstaining was performed using Mayer’s hematoxylin, and for some subsequent PAS staining was used. For RECA-1, frozen sections were used and processed via the normal IHC procedure with an AEC counterstaining. Appropriate isotype and PBS controls were consistently negative.

### Analysis of histopathological changes

Kidney sections were scanned using an Aperio Scanscope GS (Aperio Technologies, Vista, CA, USA). The extent of proximal tubular damage (Kim-1 and OPN), glomerular podocyte stress (desmin), fibrotic changes (α-SMA, Collagen I and III) (excluding vessels) was quantified using computer-assisted analysis with Aperio Imagescope (Version 9.1, Aperio, Vista, CA, USA). For α-SMA, OPN, Kim-1 and Collagen I and III, the ratio between positive pixels in the cortex and the total cortical surface area was used. For desmin the ratio between glomerular positive pixels and total cortical glomerular area was calculated. To obtain a measure of inflammation, the number of interstitial, periglomerular and glomerular macrophages (ED1-positive cells) was counted manually using light microscopy and a mean score of thirty randomly selected renal cortical fields or fifty glomeruli, both per animal, was obtained. For LVs, thirty renal cortical fields were selected and the lymphatic vessel density (LVD) was quantified by counting manually the podoplanin-positive interstitial vascular profiles per medium-power field. After excluding glomeruli and large vessels the mean percentage of RECA-1 positive peritubular capillaries per five cortical areas was determined with Image J (Image J 1.46r, National Institutes of Health, Bethesda, MD). Histopathological analysis was performed in a blinded fashion.

### Immunoblot analysis

Protein lysates were prepared from tissue cryosections (10 times, 10 μm) in RIPA buffer (Thermo Scientific) supplemented with 1% protease inhibitor cocktail (Sigma-Aldrich). Lysates were homogenized using sonication. Protein concentrations were determined using a DC protein assay (Bio-Rad, Hercules, CA, USA) after which 8μg of protein was spotted in triplicate on a Tris buffered saline (TBS) pre-soaked nitrocellulose membrane using a 96-well dot-blot apparatus (Bio-Rad). To reduce non-specific binding, spotted membranes were blocked in TBS + 0.5% Tween20 (Sigma-Aldrich) containing 5% milk powder. Spotted membranes were stained with primary antibodies directed against Collagen type I (1:1000, ab93095, Abcam) or Collagen type III (1:2000, ab6310, Abcam) diluted in TBS-Tween20 containing 5% milk powder. All washing steps were performed in TBS-Tween20. Protein bands were visualized using chemiluminescence and a ChemiDoc imaging system (Bio-Rad). Image analysis was performed using the Dot Blot analyser plugin for ImageJ, whereby collagen expression of control rats at time of biopsy was used as a reference.

### Statistical analysis

Data are expressed as the mean ± standard error of the mean (SEM). Statistical significance was accepted at p<0.05. Statistical analyses were performed using Mann-Whitney U tests or, when testing for repeated measurements, two-way ANOVA tests. For correction of multiple comparisons a Bonferroni post-hoc analysis was performed. Data were analyzed and graphed using GraphPad Prism (Version 5.00, GraphPad, San Diego, CA, USA).

## Results

### Rat characteristics – body weight, systolic blood pressure and sodium excretion

**Fig 1:**
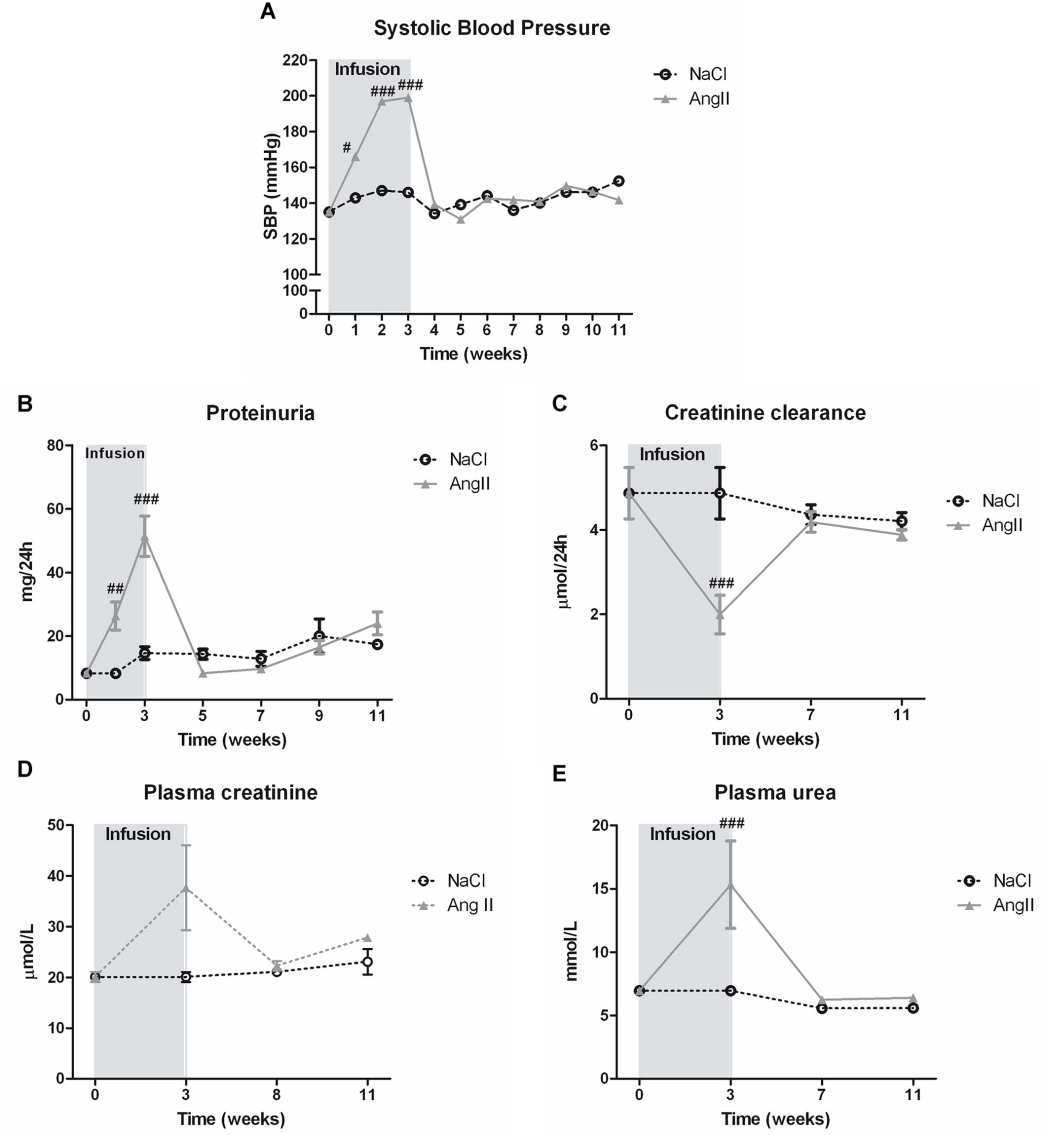
Ang II induces hypertension, renal function loss and proteinuria with spontaneous recovery after Ang II withdrawal. Ang II infusion increased systolic blood pressure (A) and urinary protein levels (B). Furthermore Ang II infusion attenuated renal function as evidenced by a decrease in creatinine clearance (C) and an increase in plasma creatinine (D). Ang II infusion also caused an increase in plasma urea levels (E). When Ang II infusion was stopped, functional renal parameters of all rats turned to control values again. (##p<0.01, ###p<0.001 vs. control).

At baseline, there were no significant differences in body weight between Ang II infused rats and NaCl-infused controls (data not shown). Throughout the infusion period, body weight of all Ang-II infused rats was significantly reduced as compared to NaCl-infused controls (321.4 ± 18.1 grams vs. 393.3 ± 7.7 grams, p<0.001). After cessation of Ang-II infusion, rats gained weight and at the end of the study former Ang-II infused rats were significantly heavier when compared to their controls (p<0.05). Three weeks of Ang-II infusion progressively increased systolic blood pressure 1.5-fold compared to NaCl-infused rats (212 ± 10.43 mmHg vs. 146 ± 1.4 mmHg, p<0.001, Fig 1A). After withdrawal of Ang-II, blood pressure immediately returned to baseline values (139 ± 4.0 mmHg vs. 134 ± 4.7 mmHg) and all rats stayed normotensive until the end of the experiment. Infusion with Ang-II significantly reduced mRNA levels of renin compared to control (p<0.05, data not shown). Urinary sodium excretion was higher in Ang-II infused rats, however this was not significant (2.7 ± 0.2 mmol/24h vs. 2.1 ± 0.2 mmol/24h).

### Spontaneous recovery of Ang II-induced proteinuria and renal function loss

There was no difference in urinary protein levels and renal function—reflected by creatinine clearance and plasma urea—for both groups at baseline (Fig 1). Under infusion of Ang II, rats gradually developed proteinuria with a significant increase of urinary protein levels already after one week of Ang II infusion (p<0.01) and a 1.9-fold increase after three weeks (51.4 ± 6.3 mg/24h vs. 14.7 ± 2.0 mg/24h, p<0.01) (Fig 1A). After three weeks of Ang II infusion, all rats had an impaired renal function as evidenced by a 59% reduction in creatinine clearance (2.0 ± 0.5 mL/min vs. 4.9 ± 0.6 mL/min, p<0.001) (Fig 1B) and a 1.9-fold increase in plasma creatinine levels (37.7 ± 8.4 μmol/L vs. 20.1 ± 1.0 μmol/L, not significant) (Fig 1C). Furthermore, Ang II infusion caused a 2.2-fold increase in plasma urea levels compared to control rats (15.3 ± 3.5 mmol/L vs. 7.0 ± 0.3 mmol/L, p<0.001) (Fig 1D). Withdrawal of Ang II treatment resulted in a spontaneous and full recovery of proteinuria, creatinine clearance and plasma urea levels.

### Effects of Ang II on tubular damage and glomerular desmin expression

**Fig 2:**
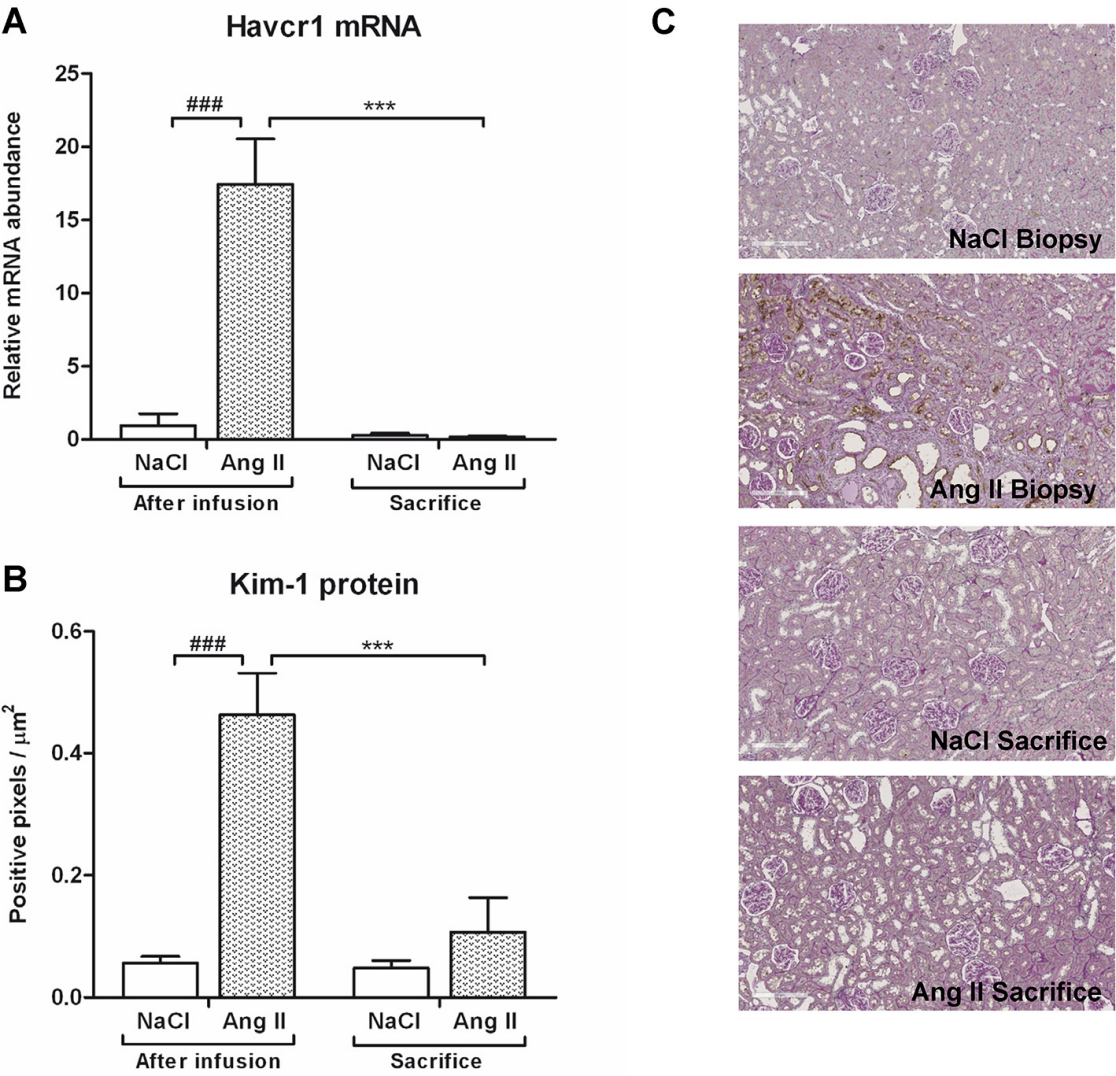
Effects of Ang II infusion and withdrawal on tubular damage. Havcr1 mRNA (A) and protein (B) levels were increased by Ang II infusion. After stopping Ang II infusion, recovery of proximal tubular damage was established, evidenced by control levels of Havcr1 mRNA and Kim-1 protein levels in Ang II infused rats. Representative photomicrographs of Kim-1 stained renal sections (C). (### p<0.001 vs. control at time of biopsy; ***p<0.001 vs. Ang II at time of biopsy).

**Fig 3:**
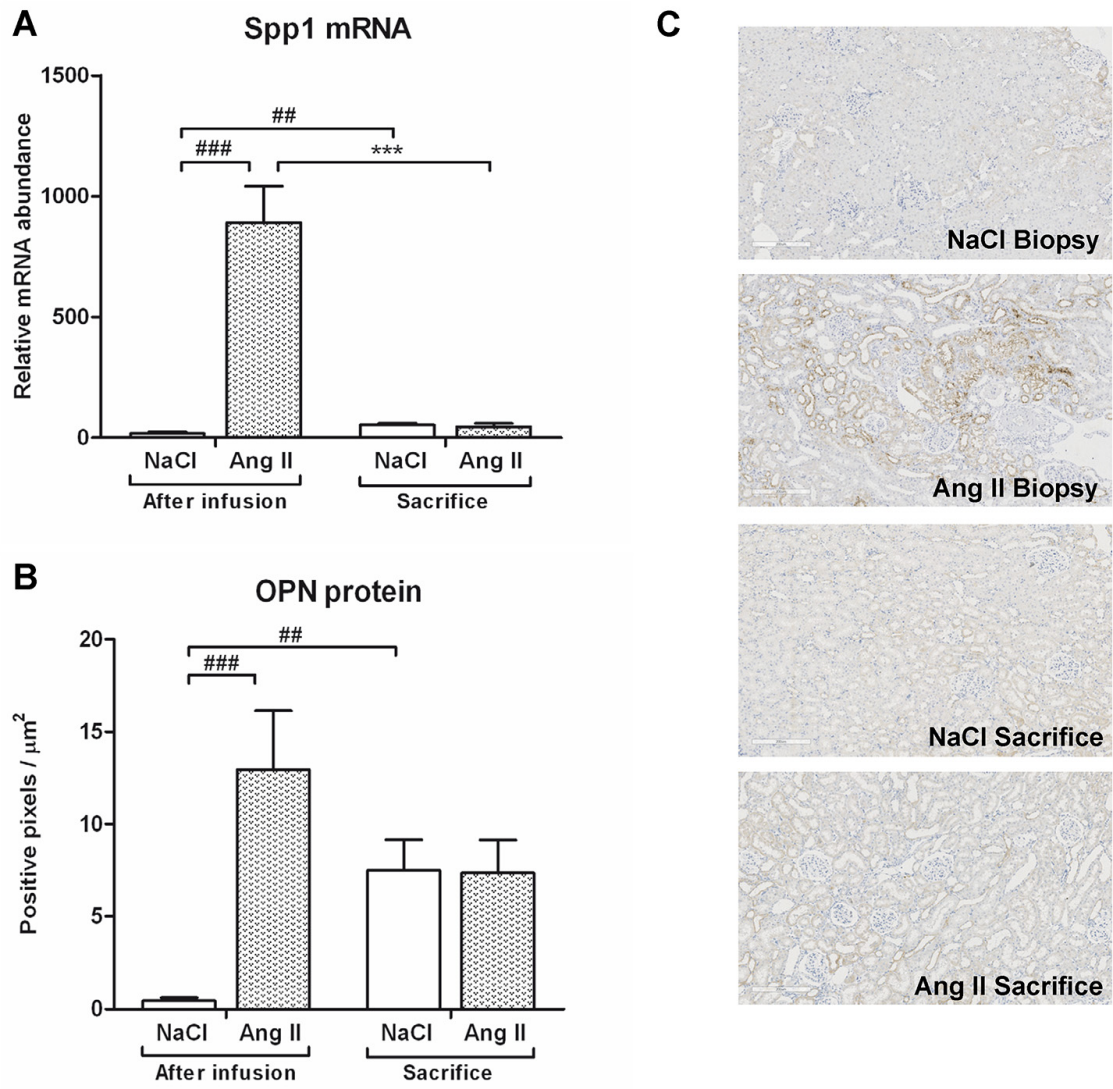
Effects of Ang II on OPN expression. Spp1 mRNA (A) and OPN protein (B) levels were increased by Ang II infusion. After stopping Ang II infusion Spp1 mRNA and OPN protein levels returned to control levels. In time, OPN protein expression of control rats significantly increased. Representative photomicrographs of OPN stained renal sections (C). (## p<0.01, ### p<0.001 *vs*. control at time of biopsy; ***p<0.001 *vs*. Ang II at time of biopsy).

**Fig 4:**
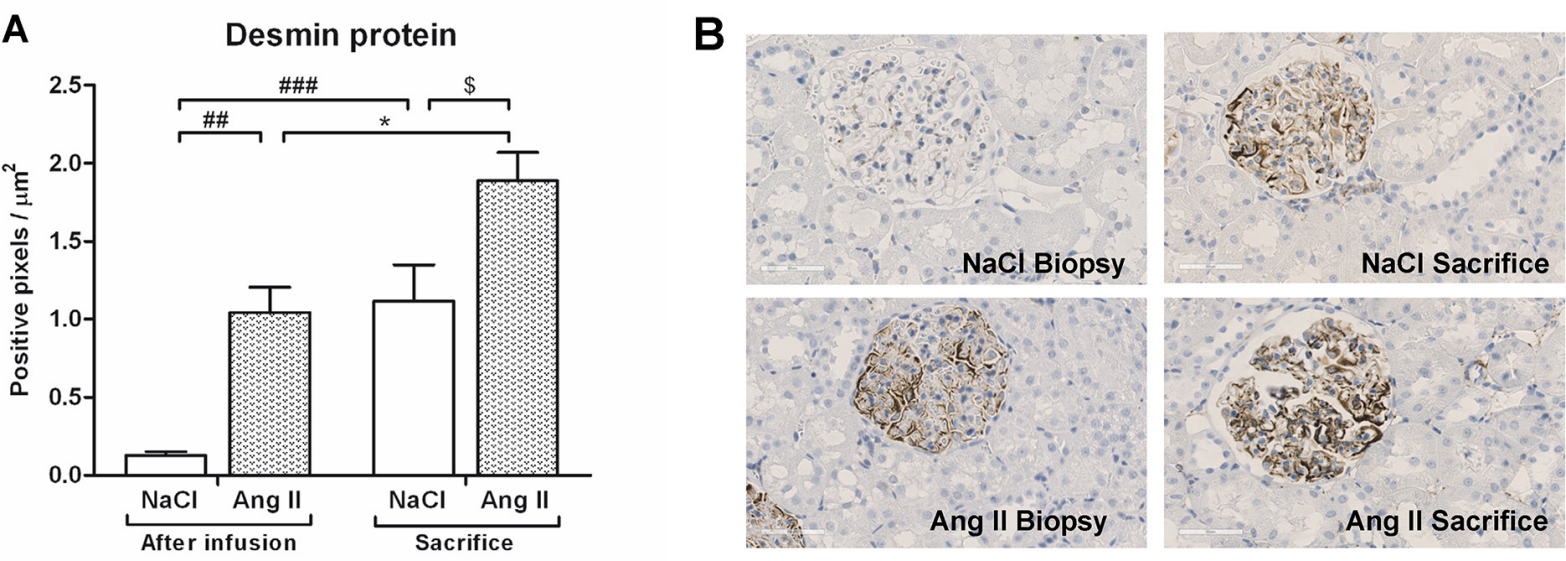
AngII infusion causes persistent glomerular desmin expression. Ang II infusion increased desmin protein levels (A). At sacrifice there was a significant increase of desmin protein in former AngII-infused rats and surprisingly in control rats as well. Compared to control rats, glomerular desmin expression of AngII-infused rats was significantly higher after stopping AngII infusion. Representative photomicrographs of desmin stained renal sections (B). (## p<0.01, ### p<0.001 *vs*. control at time of biopsy; *p<0.05 *vs*. AngII at time of biopsy; $ p<0.05 *vs*. control at time of sacrifice).

**Fig 5:**
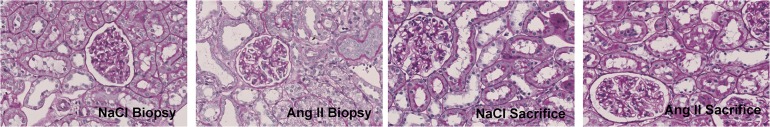
No focal segmental glomerular sclerosis after AngII infusion. Representative photomicrographs of PAS stained renal sections (A).

Ang II infusion induced tubular dilatation and atrophy as reflected by a significant increase in Havcr1 mRNA and KIM-1 protein expression (p<0.001) (Fig 2A and 2B) and Spp1 mRNA and OPN protein expression (p<0.001 and p<0.001) (Fig 3A and 3B) when compared to control rats. A complete reversibility of tubular damage was seen eight weeks after cessation of Ang II, evidenced by a reduction to control levels of both Havcr1 mRNA and KIM-1 protein expression (p<0.001) as well as SPP1 mRNA expression (p<0.001). Control rats showed a slight increase in SPP1 mRNA (p<0.01) and OPN protein (p<0.05) expression over the course of the study. At time of sacrifice, there was no difference in OPN protein expression between Ang II-infused rats and control rats. Glomerular desmin protein expression was increased 7.4-fold after three weeks of Ang II infusion (p<0.01) (Fig 4A). There was no recovery of desmin expression after cessation of Ang II infusion, and desmin protein expression of rats previously subjected to Ang II infusion even increased over the weeks when compared to their biopsy levels (p<0.05). Glomerular desmin expression of NaCl-infused rats also progressed over the weeks (p<0.001). At time of termination, Ang II-infused rats still had significantly higher levels of desmin protein expression when compared to control rats (p<0.05). Three weeks of Ang II infusion did not result in focal segmental glomerular sclerosis (Fig 5).

### Decreased interstitial but increased periglomerular and glomerular macrophage influx after cessation of Ang II infusion

**Fig 6:**
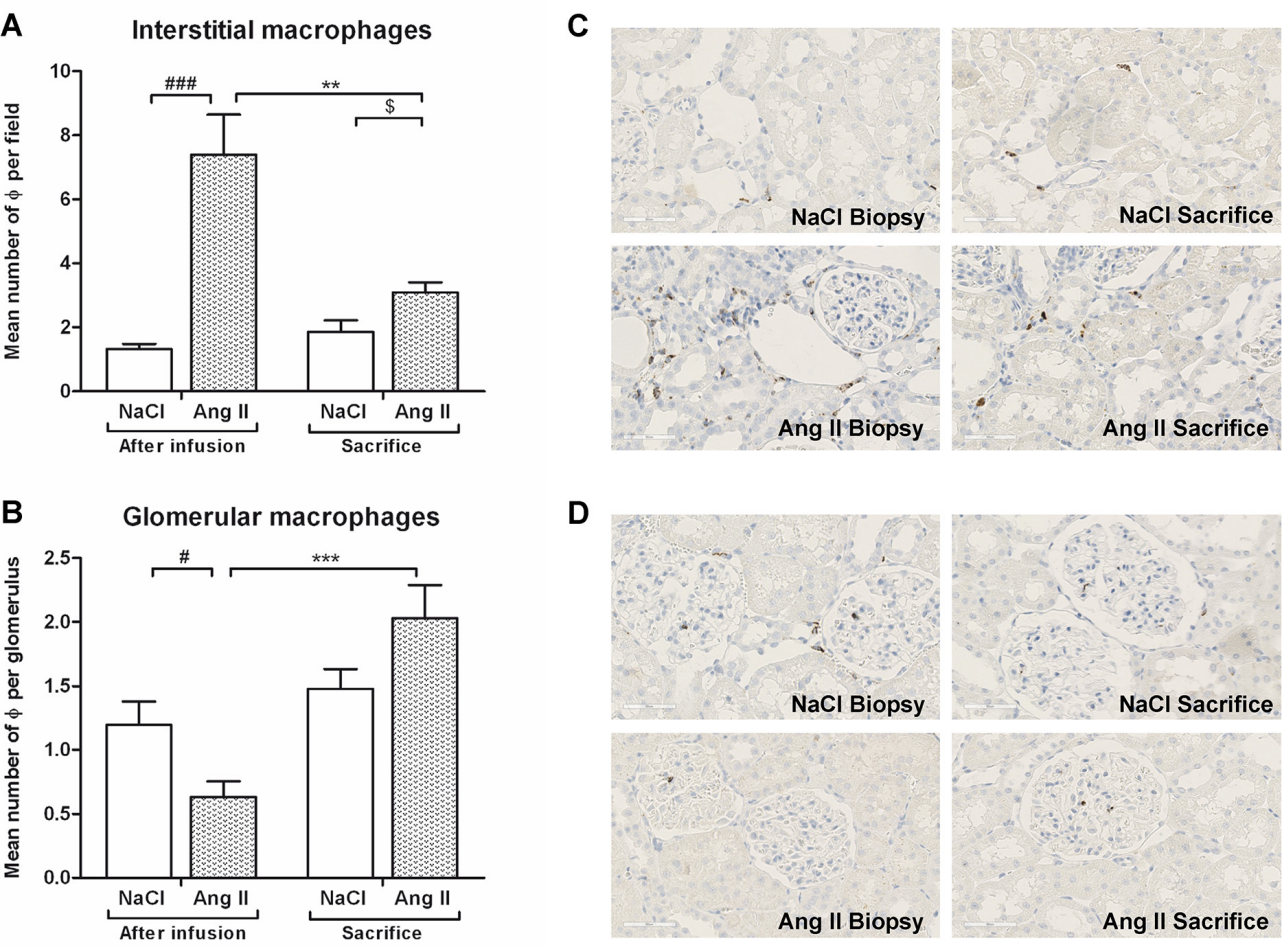
Decreased interstitial inflammation, increased periglomerular and glomerular macrophages after cessation of Ang II. Ang II increased the influx of interstitial and periglomerular macrophages. After Ang II withdrawal, the number of interstitial ED1 positive cells decreased, but was still elevated versus control rats (A). Periglomerular macrophages slightly increased after Ang II cessation, however this was not signifcant (B). For glomerular macrophages the opposite trend was found, with a decrease of macrophages per glomerulus after Ang II. After stopping infusion, Ang II-infused rats showed an increase in glomerular macrophages (C). Representative photomicrographs of ED-1 positive cells in interstitium (D) and in and around glomeruli (E). (# p<0.05, ## p<0.01, ### p<0.001 vs. control at time of biopsy; **p<0.01, ***p<0.001 vs. Ang II at time of biopsy; $ p<0.05 vs. control at time of sacrifice).

As expected, Ang II infusion increased the number of ED1 positive cells in the renal interstitium compared to controls (5.2-fold increase; 18.8 ± 4.8 vs. 3.6 ± 0.5, p<0.001) (Fig 6A). After cessation of Ang II infusion, the number of interstitial macrophages declined (p<0.01) but was still elevated when compared to control (p<0.05). Ang II infusion also significantly increased periglomerular macrophage numbers (2.91 ± 0.3 vs. 1.7 ± 0.2, p<0.01), which slightly increased after stopping Ang II infusion (Fig 6B). This was in contrast to macrophage influx in glomeruli, which significantly decreased under Ang II infusion (0.6 ± 0.1 vs. 1.2 ± 0.2, p<0.05), but significantly increased eight weeks after cessation of Ang II treatment (2.0 ± 0.3 vs. 1.5 ± 0.2, p<0.001) (Fig 6C).

### Interstitial changes after Ang II infusion, moderate recovery

**Fig 7:**
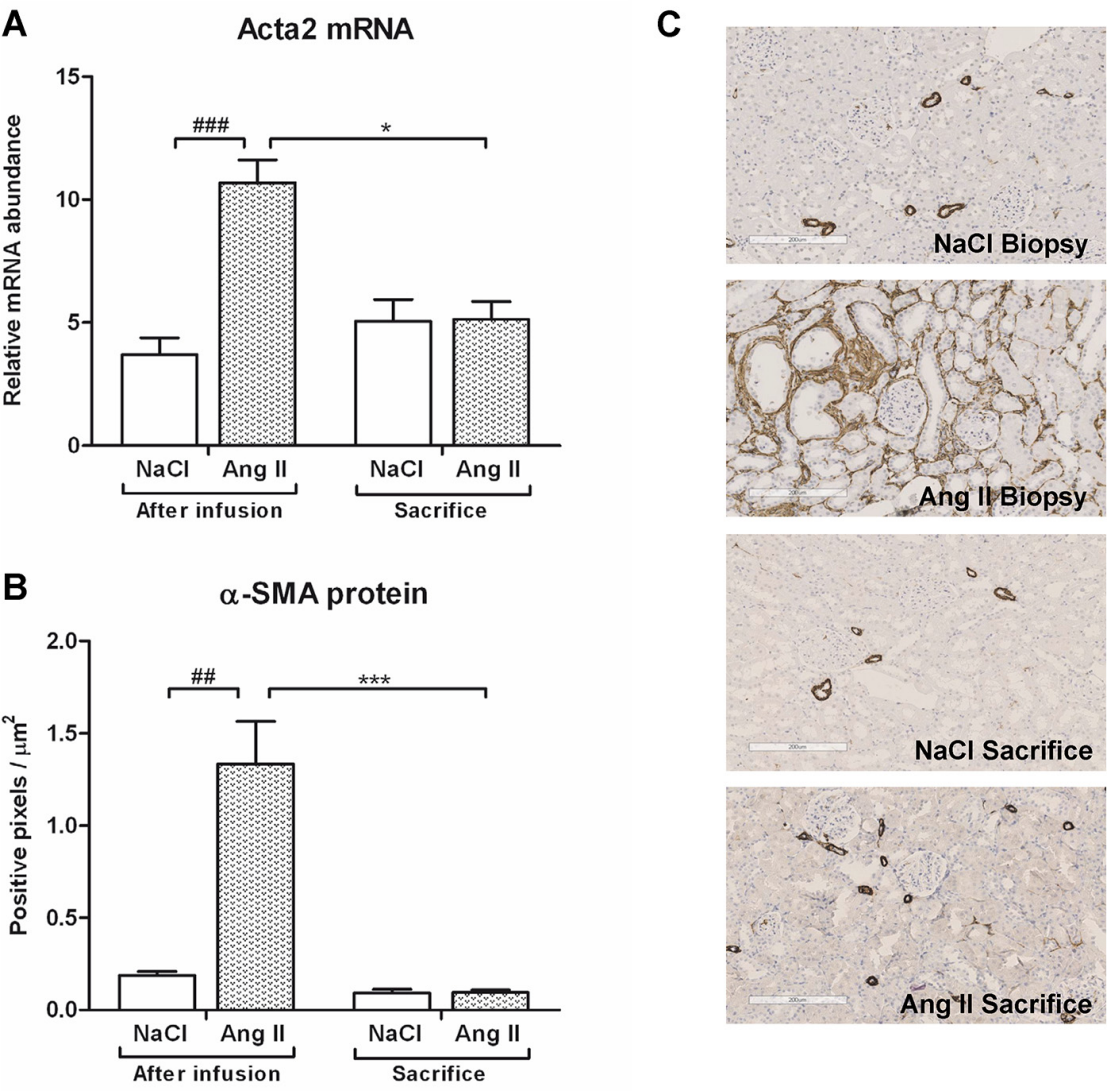
Complete reversion of Ang II-induced pre-fibrotic changes. Ang II infusion increased α-SMA mRNA (A) and protein (B) expression. At time of sacrifice, mRNA levels of Ang II-infused rats returned to control values. The same pattern holds through for α-SMA protein expression, with a complete reversion of pre-fibrotic changes after cessation of Ang II. Representative photomicrographs of α-SMA stained renal sections (C). (##p<0.01, ###p<0.001 vs. control at time of biopsy; *p<0.05, ***p<0.001 vs. Ang II at time of biopsy).

**Fig 8:**
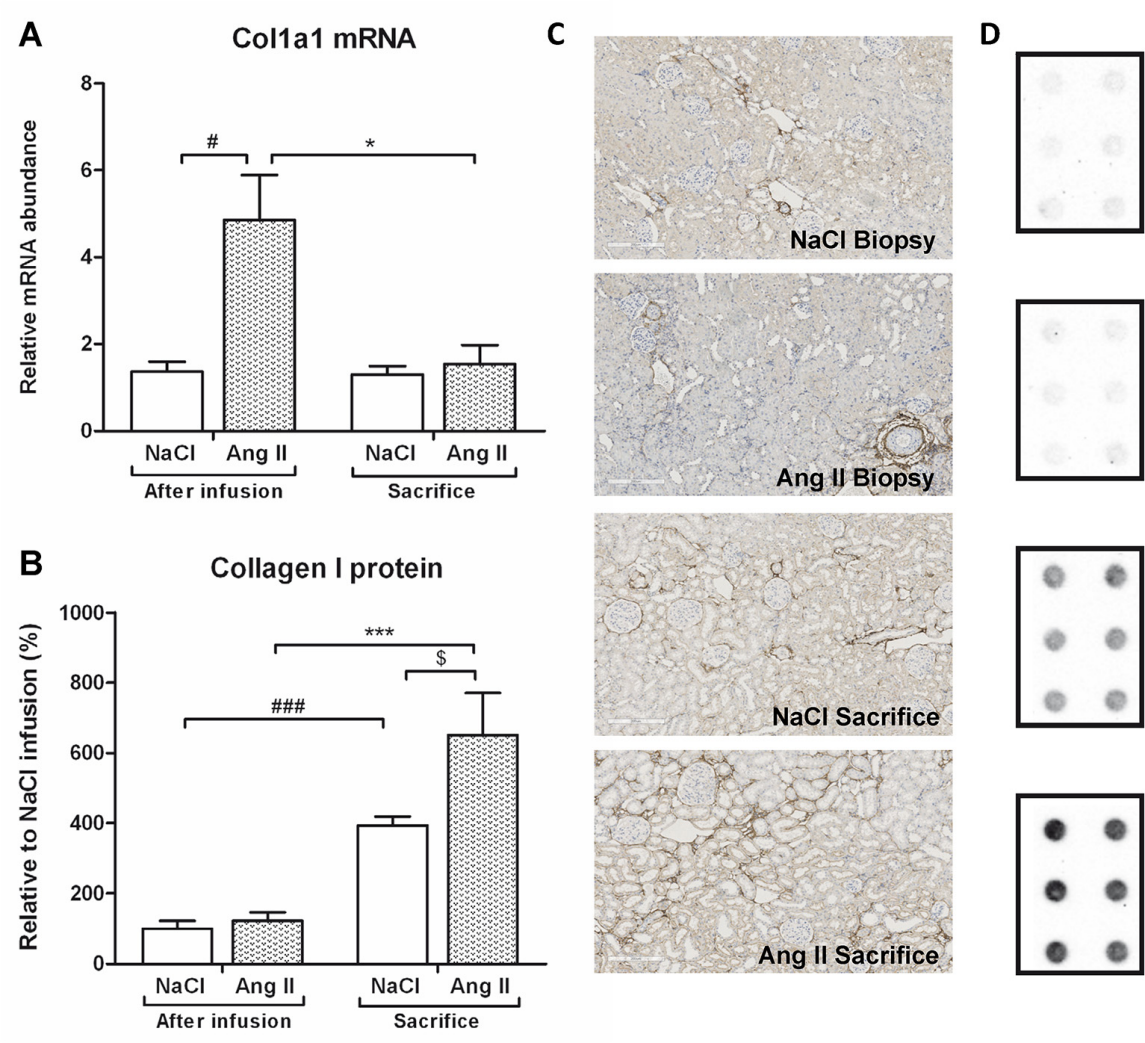
Decrease in Col1a1 mRNA expression after stopping Ang II infusion, ongoing deposition of Collagen I proteins. Ang II infusion increased Col1a1 mRNA (A) expression. After withdrawal of Ang II, Col1a1 mRNA levels returned to control values. Protein expression of Collagen I, as measured by dot blot analysis, was unaffected after infusion with Ang II. However, eight weeks after cessation of Ang II protein levels of Collagen I increased (B). Collagen I protein expression of control rats also significantly increased at sacrifice compared to their own biopsy levels (B). At time of sacrifice, former AngII-infused rats had significantly higher Collagen I protein levels when compared to control (B). Representative photomicrographs of Collagen I stained renal sections (C). Representative examples of immunoblot analysis of Collagen I of two tissue samples in triplicate (D). (#p<0.05, ###p<0.001 vs. control at time of biopsy; *p<0.05, ***p<0.001 vs. AngII at time of biopsy, $<0.05 vs. control at time of sacrifice).

**Fig 9:**
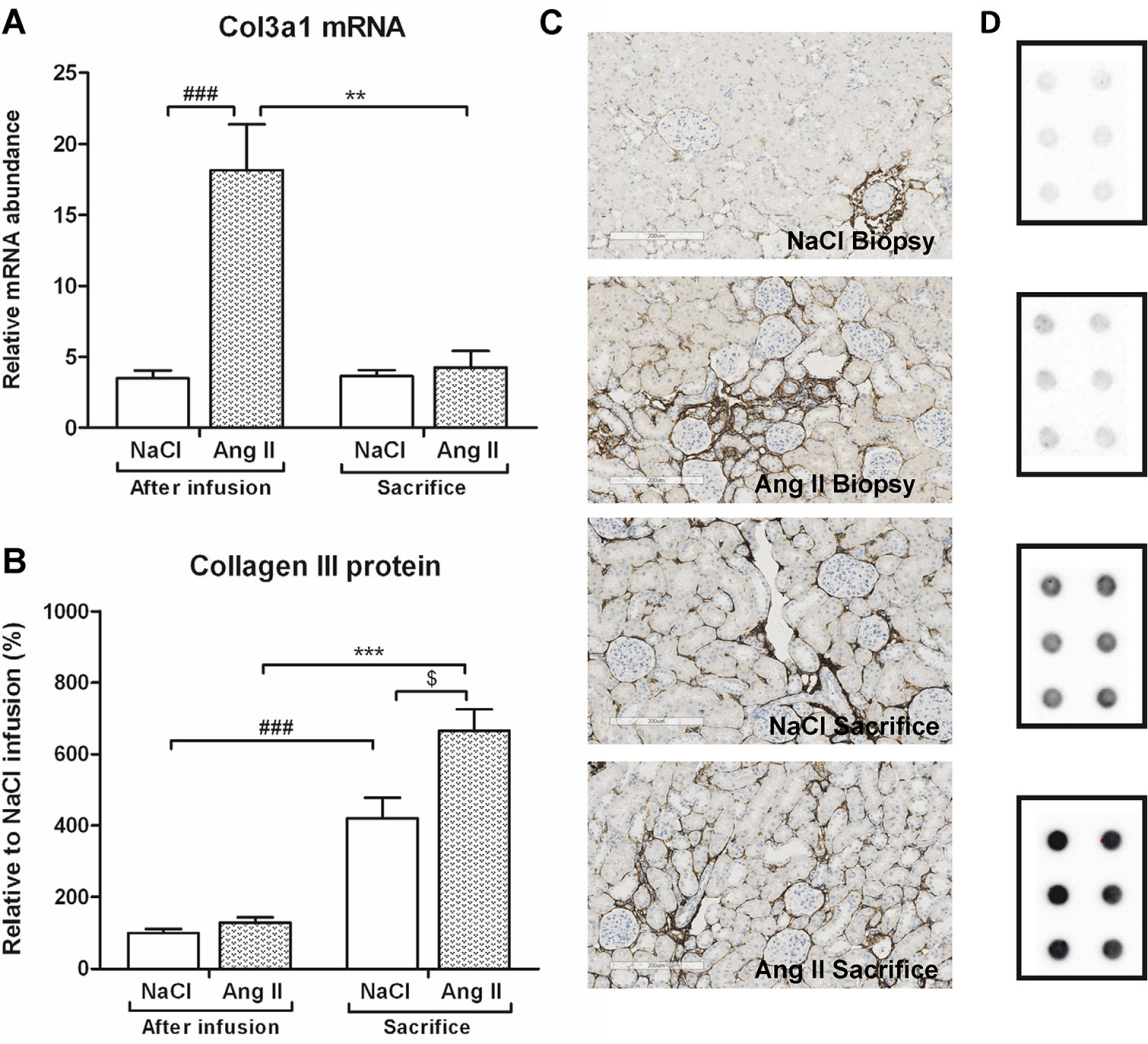
Decreased Col3a1 mRNA expression after stopping Ang II infusion, increased Collagen III protein levels. Col3a1 mRNA (A) and Collagen III protein (B) expression was increased under Ang II infusion. Eight weeks after stopping infusion with Ang II, mRNA levels of Col3a1 decreased to control levels. Protein expression of Collagen III was unaffected after infusion with Ang II, however eight weeks after cessation of Ang II protein levels of Collagen III increased (B). Collagen III protein expression of control rats also significantly increased at sacrifice compared to their own biopsy levels (B). At time of sacrifice, former AngII-infused rats had significantly higher Collagen III protein levels when compared to control (B). Representative photomicrographs of Collagen III stained renal sections (C). Representative examples of immunoblot analysis of collagen III of two tissue samples in triplicate (D) (###p<0.001 vs. control at time of biopsy; **p<0.01, ***p<0.001 vs. Ang II at time of biopsy, $p<0.05 vs. control at time of sacrifice).

**Fig 10:**
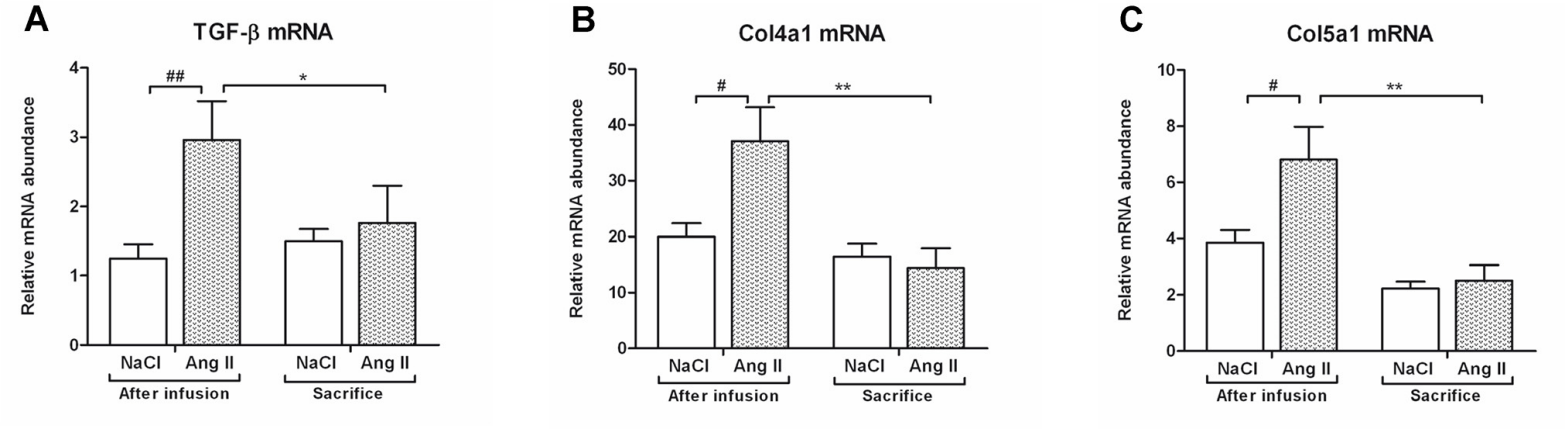
Normalization of mRNA expression of fibrotic markers after cessation of Ang II infusion. mRNA expression of the fibrotic markers TGF-beta, Col4a1 and Col5a1 was upregulated under Ang II infusion (A, B, C). Eight weeks after stopping Ang II infusion, mRNA levels of all markers returned to control levels again. (#p<0.05, ##p<0.01 *vs*. control at time of biopsy; *p<0.05, **p<0.01 *vs*. Ang II at time of biopsy).

Ang II infusion induced interstitial changes, characterized by increased mRNA and protein expression of the pre-fibrotic marker α-SMA (p<0.001) (Fig 7A and 7B). Eight weeks after cessation of Ang II infusion, levels of Acta2 mRNA (p<0.05) and protein (p<0.001) expression had reverted back to control levels. This Ang II-induced interstitial myofibroblast transformation was accompanied by increased fibrotic damage. Both Col1a1 (p<0.05) and Col3a1 (p<0.001) mRNA expression was upregulated under Ang II-infusion (Figs 8A and 9A). Eight weeks after stopping Ang II infusion mRNA expression of both collagens returned to the same level as their controls. Protein expression of Collagen I and II did not increase after three weeks of Ang II infusion (Figs 8B and 9B). Immunohistochemical and dotblot analysis showed an increased formation of Collagen I and III proteins after withdrawal of Ang II infusion (p<0.05) (Figs 8B and 9B). Control rats also showed an increase in Collagen I and Collagen III (p<0.05) protein expression over the weeks (Figs 8B and 9B). In line with Ang II-induced upregulation of Col1a1 and CoI3a1 mRNA expression, also mRNA expression of TGF-β (p<0.01), Col4a1 (p<0.05) and Col5a1 (p<0.05) mRNA was significantly increased under Ang II infusion. Eight weeks after stopping Ang II infusion, expression levels of TGF-β, Col4a1 and Col5a1 mRNA were decreased to control (Fig 10A, 10B and 10C).

### Regression of Ang II-induced lymph vessel formation

**Fig 11:**
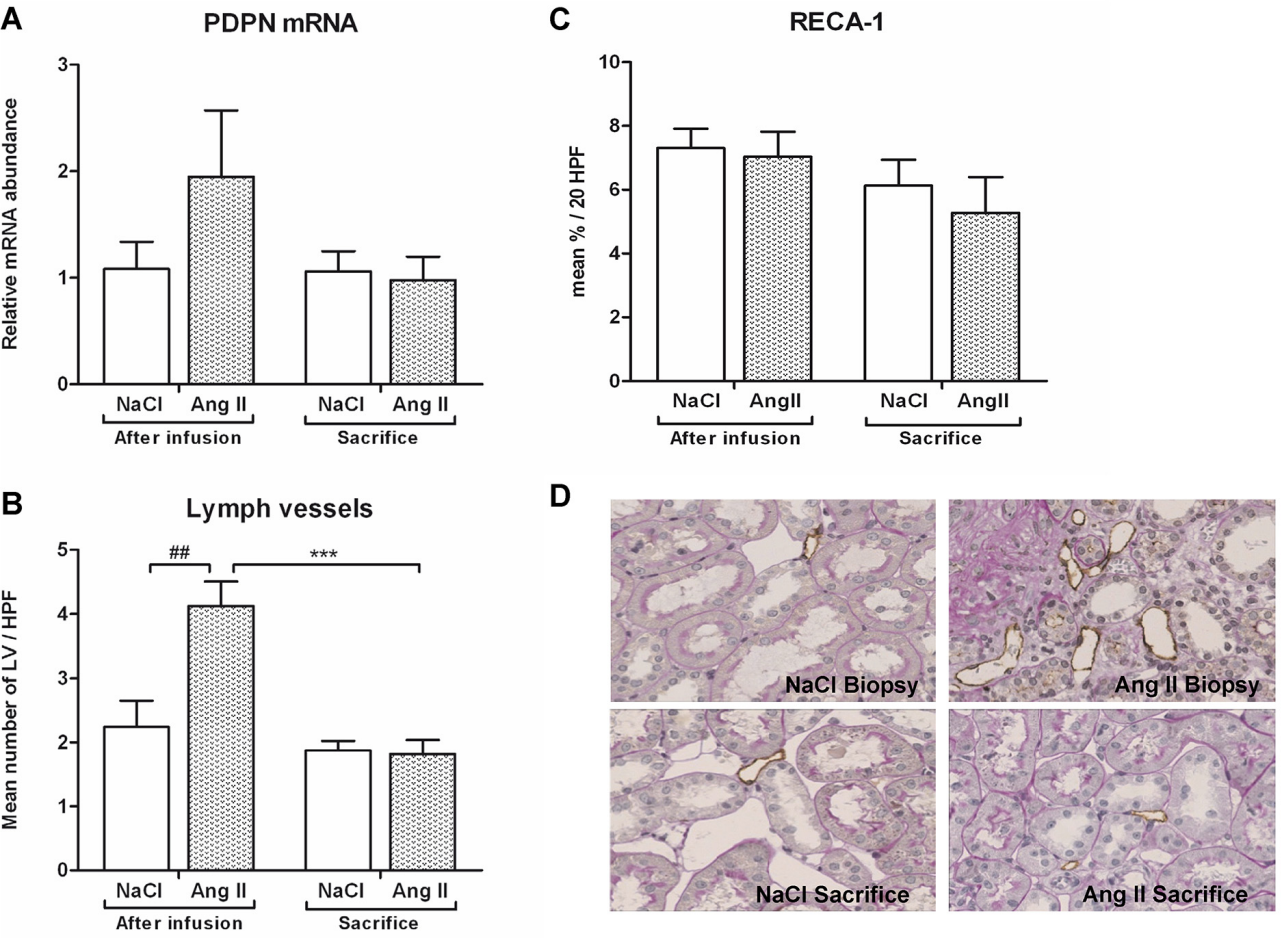
Regression of lymph vessels after Ang II withdrawal, no effect on peritubular capillaries. Ang II infusion increased mRNA expression of PDPN, however this was not significant (A). Ang II-induced lymphangiogenesis was evidenced by an increase in lymph vessel formation (B), with spontaneous regression to control levels eight weeks after withdrawal of Ang II. Ang II infusion had no effects on peritublar capillaries (C). Representative photomicrographs of Podoplanin stained renal sections (D). (##p<0.01 vs. control at time of biopsy; ***p<0.001 *vs*. Ang II at time of biopsy)

Under Ang II infusion the mRNA expression level of PDPN, a marker of lymphatic vessels (LV), was upregulated; however this was not significant (Fig 11A). Stopping Ang II infusion resulted in a decrease of PDPN mRNA expression to control levels. Ang II infusion induced a 2-fold increase in the number of LVs (4.1 ± 0.4 vs. 2.2 ± 0.4, p<0.01) (Fig 11B). At termination, the number of LVs of former Ang II infused rats regressed to control levels (p<0.001). Ang II infusion had no effects on the number of peritubular capillaries (Fig 11C).

## Discussion

The key finding of the current study is that following transient renal function decline induced by Ang II infusion, all functional parameters–blood pressure, kidney function and proteinuria–spontaneously recover within one week and remain normal throughout the follow-up period of eight weeks. At the structural level, we noted the complete reversibility of tubular damage and changes associated with myofibroblast transformation (α-SMA expression). Furthermore, we are the first to show that newly formed lymph vessels in the renal interstitium are able to undergo spontaneous regression. However, glomerular desmin expression and thin interstitial collagen fibrils remained elevated in the kidney as shown by immunohistochemical and dotblot analysis. This was accompanied by an increased number of glomerular and periglomerular macrophages. This discrepancy between functional and renal tissue parameters indicates that subclinical renal damage can persist long after removal of the triggering stimulus despite seemingly complete functional recovery.

After withdrawal of Ang II, blood pressure quickly returned to control values and rats remained normotensive until the end of the experiment. In previous studies it was found that transient exposure to Ang II can lead to the development of a renewed or sustained hypertension [16,17]. The development of salt-sensitive hypertension was described after short-term exposure of Sprague Dawley rats to Ang II when animals were subsequently exposed to a high salt diet [18]. However, in the same study rats placed on a low salt diet remained normotensive, comparable with the results described in the current study. The absence of hypertension in our model provides us with the opportunity to study renal residual changes after a temporary insult.

While others have demonstrated that two weeks of Ang II infusion at a lower dose is sufficient to induce renal damage [19,20], we here infused Ang II at a dose of 435 ng/kg/min for the duration of three weeks. This might explain the relatively high levels of hypertension, urinary protein excretion and degree of renal inflammation and collagen deposition in the present study. However, the data is consistent with a previous publication from our group using the same dose and infusion time of Ang II in Sprague-Dawley rats [21].

Both in rats and in humans renal functional and structural damage spontaneously develops as a consequence of aging [3,22–27]. In the current experiment, we potentially found slight effects of aging with a decrease in creatinine clearance and an increase in urinary protein excretion, plasma urea levels, glomerular desmin protein expression and interstitial collagen protein expression of control rats at time of termination when compared to their own baseline values. It was demonstrated that in Wistar rats, glomerular changes and proteinuria gradually develop as a consequence of aging [28]. However, aging alone might not be sufficient to explain the findings of increased glomerular desmin and interstitial collagen deposition in our control rats. An additional explanation for renal functional decline and glomerular and interstitital changes over the weeks can be induced by the implementation of the osmotic minipump and/or the kidney biopsy procedure.

An incomplete resolution of Ang II-induced renal functional and structural changes, including evidence of injury and higher levels of serum creatinine after cessation of Ang II administration, has been shown before [18]. We now demonstrate that despite the fact that urinary protein levels return to baseline values, podocyte stress as evidenced by glomerular desmin expression [29], and increased influx of periglomerular and glomerular inflammatory cells is still present eight weeks after cessation of Ang II. The glomerular deposition of desmin and intraglomerular inflammation can cause foot process fusion, with an increased risk of redeveloping proteinuria [30,31]. The relevance of periglomerular infiltrates is substantiated by Heymann et al. who found that induced periglomerular mononuclear infiltrate was associated with parietal podocyte damage [32]. Furthermore, we previously demonstrated that depletion of glomerular macrophages in a rat renal ablation model leads to decreased glomerular mesangial expansion, indicating that glomerular macrophages are involved in the initiation and progression of glomerular damage [33]. Whether or not the low numbers of glomerular macrophages in the present model are of biological significance is unknown.

In the present study hard endpoints such as FSGS and interstitial fibrosis were absent in PAS stained renal sections. Renal damage consisted of influx of inflammatory cells, glomerular desmin expression and accumulation of extracellular matrix. The increased glomerular desmin expression in the present study is in line with enhanced collagen deposition. We demonstrated that after three weeks of Ang II infusion mRNA expression of collagens is significantly increased, which was followed by an increased protein expression at the end of the experiment, despite cessation of Ang II infusion. Of course, it takes time for collagen deposition to occur; alternatively, this discrepancy may be due to insufficient enzymatic degradation of collagens in the first phase, whereas the remaining interstitial collagen deposition towards the end of the experiment is a consequence of the formation of insoluble collagen crosslinks, with subsequent extracellular matrix (ECM) formation [34,35]. When the remaining fibrotic ECM is exposed to de novo stimuli including aging related changes, the extent of defense and repair of this damage is incomplete and likely to accumulate over time. This has been shown to result in structural and functional changes, with significant effects on cell adhesion, proliferation and also altered cell signaling as a consequence [36]. Cytokines, growth factors and reactive oxygen species in combination with fibrotic ECM formation are likely to be of great significance in the development and progression of CKD [37]. Moreover, fibrotic ECM is known to function as a docking platform for fibrogenic stimuli [38].

Lymph vessel formation is a key event in renal interstitial fibrosis [39]. Inflammation-induced lymphangiogenesis is described in multiple human disease conditions [40–43] as well as in animal studies [44,45]. We are the first to show that newly formed renal lymph vessels can undergo spontaneous regression. In contrast to findings of persisting lymphatic vessels in a model of chronic airway inflammation [41], we found that eight weeks after cessation of Ang II both Podoplanin mRNA and protein expression were back to control levels. In pulmonary fibrosis it has been shown that Ang II contributes to progression of the disease [46] and that both Angiotensin II type 1 and 2 receptors and LVs play an important role in organ remodeling and fibrosis [47]. In the kidney, lymphangiogenesis occurs after established proteinuria and prior to collagen deposition, fibrosis and macrophage influx in a rat model of unilateral adriamycin nephrosis and ACE inhibition significantly prevents this new lymph vessel formation [39,48]. In addition, one of the commonly found events in proteinuria-associated diseases is renal interstitial oedema and swelling, and this has been shown to be an important factor in provoking lymphangiogenesis [39]. It is possible that during the process of renal remission growth factors involved in stimulating lymphangiogenesis such as VEGF-C and D [49] are absent, eventually leading to lymphatic endothelial cell apoptosis and LVs regression. Despite dynamic changes in lymph vessel formation, the number of peritubular capillaries was unaltered in our model.

A decreased expression of the proinflammatory cytokine OPN is presumably a consequence of normalization of the blood pressure and proteinuria in our experiment. OPN plays a crucial role in the development of Ang II-induced renal damage [50,51] by inducing inflammation, oxidative stress and fibrosis [50]. Interestingly, it has been shown recently that OPN can also directly induce lymphangiogenesis [52]. Ang II infusion increased OPN mRNA and protein expression, and eight weeks after cessation of Ang II renal OPN expression levels were down back to control levels. A decrease in OPN expression not only leads to renal structural improvement, but is also accompanied by enhanced renal function [53]. Suppression of OPN expression, by e.g. IL-1 receptor antagonist treatment or RAAS inhibition, reversed OPN-induced loss of renal function, macrophage influx and severe histological damage [54,55]. All these results indicate that the decreased level of OPN expression might contribute to the reversibility of the Ang II-induced pre-fibrotic damage markers in our experiment.

The present study has strengths and limitations. The experimental set-up with every rat functioning as its own control, by comparing renal functional changes with renal tissue changes at time of biopsy versus termination after a follow-up period of 8 weeks, makes the study unique and powerful. Furthermore, a lot of different parameters for renal function and structural changes were determined to obtain insight into the processes of development of renal damage as well as spontaneous recovery and persisting damage. A limitation of the study is the method used to assed renal function. Creatinine clearance is widely used to assess renal function in experimental renal disease [56], however an important complications to this technique is tubular creatinine secretion, which leads to an overestimation of the real GFR to various extent also in the rat [57, 58]. Additional experiments are needed to sort out the effects of Ang II infusion on tubular creatinine excretion. The lack of inclusion of control rats for the biopsy procedure makes the interpretation of increased renal damage parameters in control rats difficult.

In conclusion, despite full restoration of renal function, renal abnormalities at the tissue level induced by Ang II infusion were only partially restored, with remaining glomerular desmin expression, increased influx of periglomerular and glomerular macrophages and interstitial collagen deposition. This incomplete tissue restoration indicates that the initiation and progression of renal damage is ongoing after the initial injury to the kidney, despite absence of clinical signs and symptoms of renal disease. Further studies are required to identify the most important players in this process and to investigate to what extent this remaining damage has consequences for long-term renal outcome.
